# 7,7-Dimethyl-2-methyl­amino-4-(4-methyl­phenyl)-3-nitro-7,8-di­hydro-4*H*-chromen-5(6*H*)-one

**DOI:** 10.1107/S1600536814010794

**Published:** 2014-05-24

**Authors:** S. Antony Inglebert, Jayabal Kamalraja, K. Sethusankar, Paramasivam T. Perumal

**Affiliations:** a Sri Ram Engineering College, Chennai 602 024, India; b Organic Chemistry Division, CSIR Central Leather Research Institute, Chennai 600 020, India; c Department of Physics, RKM Vivekananda College (Autonomous), Chennai 600 004, India

## Abstract

In the title compound, C_19_H_22_N_2_O_4_, the six-membered cyclo­hexenone ring of the chromene unit adopts an envelope conformation, with the dimethyl-substituted C atom as the flap, while the pyran ring has a boat conformation. These two mean planes are inclined to one another by 6.65 (13)°·The benzene ring is normal to the 4*H*-chromene moiety mean plane, making a dihedral angle of 89.18 (5)°. The methyl­amine and nitro groups are slightly twisted from the chromene moiety mean plane, with torsion angles C—N—C—O = 1.70 (18) and O—N—C—C = 0.15 (18)°. The mol­ecular structure is characterized by an intra­molecular N—H⋯O hydrogen bond, which generates an *S*(6) ring motif. In the crystal, mol­ecules are linked *via* pairs of weak C—H⋯O hydrogen bonds, forming inversion dimers. These dimers are connected by further C—H⋯O hydrogen bonds, forming sheets lying parallel to (10-1).

## Related literature   

For the biological and pharmacological properties of chromenes and their derivatives, see: Zonouzi *et al.* (2013 ▶). For related structures, see: Narayanan *et al.* (2013 ▶); Inglebert *et al.* (2014 ▶).
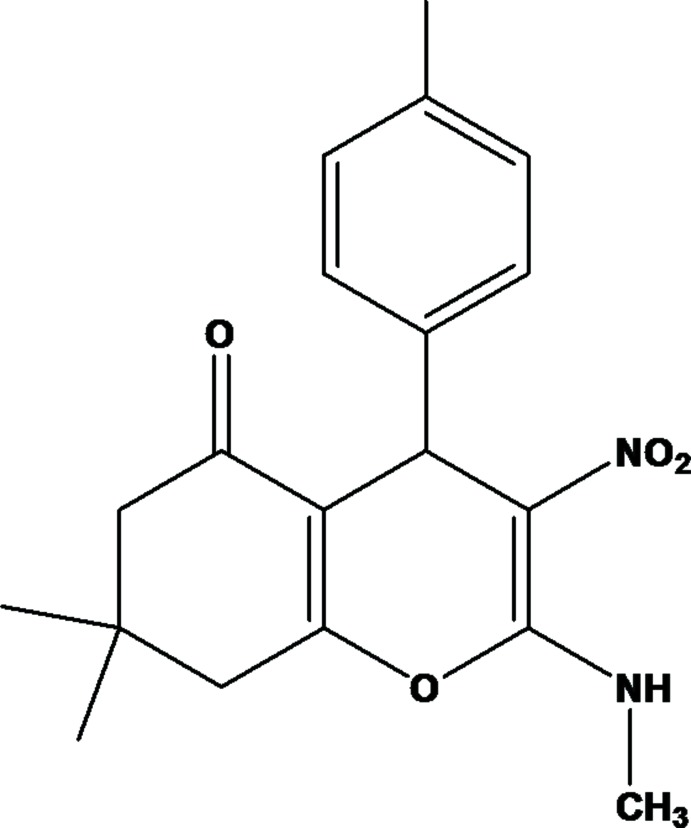


## Experimental   

### 

#### Crystal data  

C_19_H_22_N_2_O_4_
*M* *_r_* = 342.39
Monoclinic, 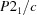

*a* = 9.4373 (5) Å
*b* = 15.8487 (8) Å
*c* = 12.1414 (6) Å
β = 105.122 (1)°
*V* = 1753.09 (15) Å^3^
*Z* = 4
Mo *K*α radiation
μ = 0.09 mm^−1^
*T* = 293 K
0.30 × 0.25 × 0.20 mm

#### Data collection  

Bruker Kappa APEXII CCD diffractometer
Absorption correction: multi-scan (*SADABS*; Bruker, 2008 ▶) *T*_min_ = 0.968, *T*_max_ = 0.968
22747 measured reflections
4023 independent reflections
3290 reflections with *I* > 2σ(*I*)
*R*_int_ = 0.022

#### Refinement  

*R*[*F* ^2^ > 2σ(*F* ^2^)] = 0.041
*wR*(*F* ^2^) = 0.129
*S* = 1.04
4023 reflections
226 parameters
H-atom parameters constrained
Δρ_max_ = 0.24 e Å^−3^
Δρ_min_ = −0.27 e Å^−3^

### 

Data collection: *APEX2* (Bruker, 2008 ▶); cell refinement: *SAINT* (Bruker, 2008 ▶); data reduction: *SAINT*; program(s) used to solve structure: *SHELXS97* (Sheldrick, 2008 ▶); program(s) used to refine structure: *SHELXL97* (Sheldrick, 2008 ▶); molecular graphics: *ORTEP-3 for Windows* (Farrugia, 2012 ▶); software used to prepare material for publication: *SHELXL97* and *PLATON* (Spek, 2009 ▶).

## Supplementary Material

Crystal structure: contains datablock(s) global, I. DOI: 10.1107/S1600536814010794/su2733sup1.cif

Structure factors: contains datablock(s) I. DOI: 10.1107/S1600536814010794/su2733Isup2.hkl

Supporting information file. DOI: 10.1107/S1600536814010794/su2733Isup3.cml

CCDC reference: 1002204

Additional supporting information:  crystallographic information; 3D view; checkCIF report

## Figures and Tables

**Table 1:** Hydrogen-bond geometry (Å, °)

*D*—H⋯*A*	*D*—H	H⋯*A*	*D*⋯*A*	*D*—H⋯*A*
N1—H1⋯O3	0.86	1.98	2.6024 (15)	129
C5—H5⋯O3^i^	0.93	2.58	3.0685 (17)	113
C10—H10*A*⋯O4^ii^	0.96	2.54	3.4335 (19)	154

Symmetry codes: (i) 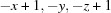; (ii) 
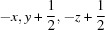
.

**Fig. 1.**
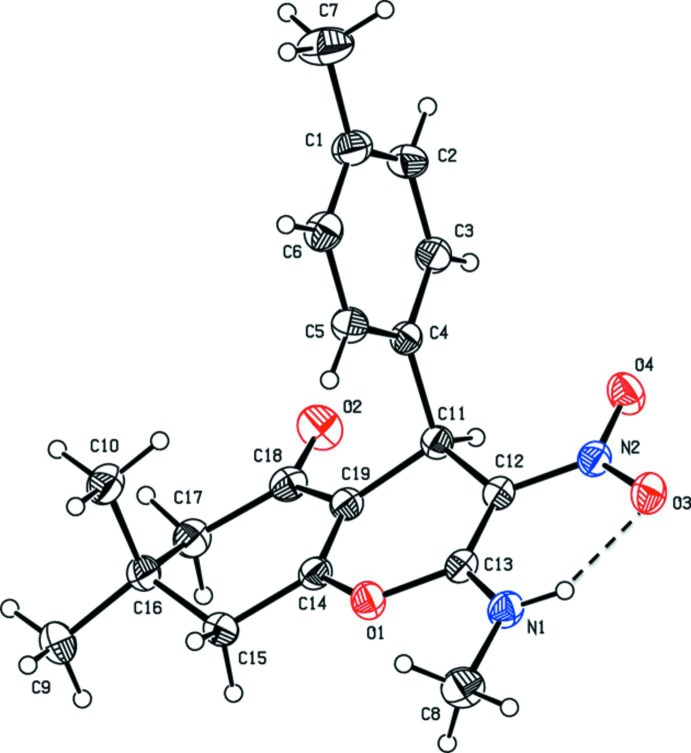
A view of the molecular structure of the title molecule, with atom labelling. Displacement ellipsoids are drawn at the 30% probability level. The intramolecular hydrogen bond is shown as a dashed line (see Table 1 for details).

**Fig. 2.**
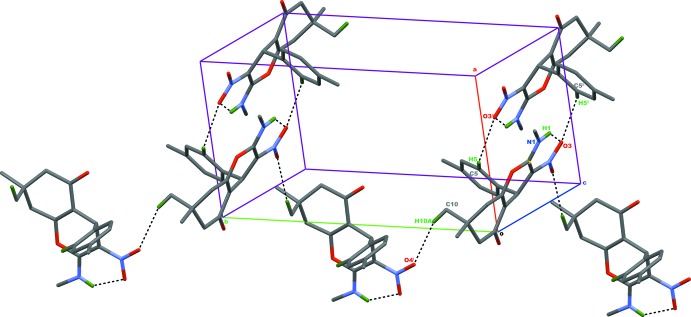
The crystal packing of the title compound viewed along the c axis. Hydrogen bonds are shown as dashed lines (see Table 1 for details; H atoms not involved in hydrogen bonding have been omitted for clarity).

## supplementary crystallographic information

### 1. Comment

4*H*-Chromene derivatives are important scaffolds in organic and medicinal chemistry. 4*H*-Chromene and their derivatives exhibit a wide spectrum of biological applications, such as antiallergic, anti-proliferative, anticancer, antibacterial, antiviral and potent apoptosis (Zonouzi *et al.*, 2013).

The title compound, Fig. 1, consists of a chromene unit connected to a toluene ring at C11, a nitro group at C12, a methyl amine group at C13, dimethyl group at C16 and an oxygen atom at C18. The benzene ring (C1-C6) is normal to the mean plane of chromene unit (O1/C11–C19) with a dihedral angle of 89.18 (5)°. The nitro and methylamine groups are inclined to the mean plane of chromene unit by 6.51 (8) and 5.42 (6)°, respectively.

The six membered carbocyclic ring (C14–C19) of the chromene moiety adopts an *envelope* conformation with atom C16 as the flap: puckering amplitude (Q) = 0.4584 (15) Å, θ = 58.54 (17)° φ = 120.3 (2)°. Atom C16 deviates by 0.3234 (15) Å from the mean plane passing through the rest of the ring atoms. This conformation is similar to that in related structures (Narayanan *et al.*, 2013; Inglebert *et al.*, 2014).

The amine group nitrogen atoms, N1 and N2, deviate by -0.2044 (11) and -0.1338 (11) Å from the mean plane of the chromene moiety. The methyl amine group attached to C13 is coplanar with the chromene moiety as indicated by the torsion angle C8–N1–C13–O1 = 1.70 (18)°. The nitro group is also coplanar to the chromene moiety mean plane, as indicated by the torsion angles C13–C12–N2–O3 = 0.15 (18)° and C11–C12–N2–O4 = -4.82 (16)°. The molecular structure is characterized by an intramolecular N—H···O hydrogen bond, generates an *S(6)* ring motif (Fig. 1 and Table 1).

In the crystal, molecules are linked via pairs of C—H···O hydrogen bonds forming inversion dimers (Fig. 2 and Table 1). These dimers are connected by further C-H···O hydrogen bonds forming sheets lying parallel to (10-1) [Table 1 and Fig. 2]. The nitro atom O3 is involved in both intra- and inter-molecular hydrogen bonding, having a bifurcated character.

### 2. Experimental

A solution of 4-methylbenzaldehyde (1.0 mmol), 5,5-dimethylcyclohexane-1,3-dione (1.0 mmol), NMSM (1.0 mmol), and piperidine (0.2 equiv) in ethanol (2 ml) was stirred for 3.5 h. After the reaction was complete, as indicated by TLC, the product was filtered and washed with 2 ml of ethanol, to remove the excess base and other impurities. Finally, the product was recrystallized from ethanol yielding colourless block-like crystals.

### 3. Refinement

H atoms were placed in idealized positions and allowed to ride on the parent atoms: N-H = 0.86 Å, and C—H = 0.93, 0.96 and 0.97 Å for aromatic, methyl and methylene H atoms, respectively, with *U*_iso_(H)= 1.5 *U*_eq_(C-methyl) and = 1.2U_eq_(C,N) for other H atoms.

### Crystal data

**Table:**

C_19_H_22_N_2_O_4_	*F*(000) = 728
*M**_r_* = 342.39	*D*_x_ = 1.297 Mg m^−^^3^
Monoclinic, *P*2_1_/*c*	Mo *K*α radiation, λ = 0.71073 Å
Hall symbol: -P 2ybc	Cell parameters from 4023 reflections
*a* = 9.4373 (5) Å	θ = 2.2–27.5°
*b* = 15.8487 (8) Å	µ = 0.09 mm^−^^1^
*c* = 12.1414 (6) Å	*T* = 293 K
β = 105.122 (1)°	Block, colourless
*V* = 1753.09 (15) Å^3^	0.30 × 0.25 × 0.20 mm
*Z* = 4	

### Data collection

**Table:**

Bruker Kappa APEXII CCD diffractometer	4023 independent reflections
Radiation source: fine-focus sealed tube	3290 reflections with *I* > 2σ(*I*)
Graphite monochromator	*R*_int_ = 0.022
ω and φ scan	θ_max_ = 27.5°, θ_min_ = 2.2°
Absorption correction: multi-scan (*SADABS*; Bruker, 2008)	*h* = −12→12
*T*_min_ = 0.968, *T*_max_ = 0.968	*k* = −20→20
22747 measured reflections	*l* = −15→15

### Refinement

**Table:**

Refinement on *F*^2^	Primary atom site location: structure-invariant direct methods
Least-squares matrix: full	Secondary atom site location: difference Fourier map
*R*[*F*^2^ > 2σ(*F*^2^)] = 0.041	Hydrogen site location: inferred from neighbouring sites
*wR*(*F*^2^) = 0.129	H-atom parameters constrained
*S* = 1.04	*w* = 1/[σ^2^(*F*_o_^2^) + (0.0725*P*)^2^ + 0.3328*P*] where *P* = (*F*_o_^2^ + 2*F*_c_^2^)/3
4023 reflections	(Δ/σ)_max_ < 0.001
226 parameters	Δρ_max_ = 0.24 e Å^−^^3^
0 restraints	Δρ_min_ = −0.27 e Å^−^^3^

### Special details

**Table:**

Geometry. All e.s.d.'s (except the e.s.d. in the dihedral angle between two l.s. planes) are estimated using the full covariance matrix. The cell e.s.d.'s are taken into account individually in the estimation of e.s.d.'s in distances, angles and torsion angles; correlations between e.s.d.'s in cell parameters are only used when they are defined by crystal symmetry. An approximate (isotropic) treatment of cell e.s.d.'s is used for estimating e.s.d.'s involving l.s. planes.
Refinement. Refinement of *F*^2^ against ALL reflections. The weighted *R*-factor *wR* and goodness of fit *S* are based on *F*^2^, conventional *R*-factors *R* are based on *F*, with *F* set to zero for negative *F*^2^. The threshold expression of *F*^2^ > σ(*F*^2^) is used only for calculating *R*-factors(gt) *etc*. and is not relevant to the choice of reflections for refinement. *R*-factors based on *F*^2^ are statistically about twice as large as those based on *F*, and *R*- factors based on ALL data will be even larger.

### Fractional atomic coordinates and isotropic or equivalent isotropic displacement parameters (Å^2^)

**Table:**

	*x*	*y*	*z*	*U*_iso_*/*U*_eq_	
C1	0.35586 (16)	0.13090 (10)	0.09850 (12)	0.0483 (3)	
C2	0.26190 (17)	0.06267 (10)	0.06696 (12)	0.0482 (3)	
H2	0.2468	0.0401	−0.0059	0.058*	
C3	0.19010 (15)	0.02757 (8)	0.14194 (11)	0.0415 (3)	
H3	0.1263	−0.0175	0.1184	0.050*	
C4	0.21229 (13)	0.05885 (7)	0.25161 (10)	0.0337 (3)	
C5	0.30488 (14)	0.12771 (8)	0.28302 (11)	0.0396 (3)	
H5	0.3197	0.1505	0.3557	0.047*	
C6	0.37535 (15)	0.16289 (9)	0.20772 (12)	0.0458 (3)	
H6	0.4371	0.2089	0.2307	0.055*	
C7	0.4372 (2)	0.16838 (15)	0.01843 (17)	0.0787 (6)	
H7A	0.4967	0.2146	0.0554	0.118*	
H7B	0.3679	0.1883	−0.0492	0.118*	
H7C	0.4987	0.1261	−0.0018	0.118*	
C8	0.41028 (17)	0.05680 (10)	0.74565 (12)	0.0526 (4)	
H8A	0.4903	0.0313	0.8011	0.079*	
H8B	0.3235	0.0552	0.7728	0.079*	
H8C	0.4343	0.1143	0.7336	0.079*	
C9	−0.19570 (18)	0.30022 (10)	0.45333 (15)	0.0582 (4)	
H9A	−0.2572	0.2675	0.4889	0.087*	
H9B	−0.2558	0.3308	0.3899	0.087*	
H9C	−0.1388	0.3392	0.5077	0.087*	
C10	0.00622 (16)	0.29312 (9)	0.35491 (13)	0.0492 (3)	
H10A	−0.0532	0.3238	0.2913	0.074*	
H10B	0.0708	0.2559	0.3287	0.074*	
H10C	0.0632	0.3320	0.4094	0.074*	
C11	0.13852 (13)	0.01918 (7)	0.33750 (10)	0.0339 (3)	
H11	0.0773	−0.0281	0.3006	0.041*	
C12	0.24902 (13)	−0.01314 (7)	0.44158 (10)	0.0343 (3)	
C13	0.28241 (13)	0.02932 (8)	0.54548 (10)	0.0352 (3)	
C14	0.08036 (14)	0.12100 (8)	0.47489 (10)	0.0362 (3)	
C15	0.00113 (15)	0.19200 (9)	0.51285 (11)	0.0425 (3)	
H15A	0.0718	0.2298	0.5609	0.051*	
H15B	−0.0615	0.1699	0.5580	0.051*	
C16	−0.09273 (14)	0.24144 (8)	0.41109 (12)	0.0406 (3)	
C17	−0.18292 (14)	0.17886 (9)	0.32523 (12)	0.0440 (3)	
H17A	−0.2584	0.1552	0.3570	0.053*	
H17B	−0.2318	0.2095	0.2567	0.053*	
C18	−0.09720 (13)	0.10749 (8)	0.29228 (11)	0.0388 (3)	
C19	0.04223 (13)	0.08322 (8)	0.37386 (10)	0.0344 (3)	
N1	0.38424 (12)	0.01069 (7)	0.63882 (9)	0.0410 (3)	
H1	0.4396	−0.0320	0.6365	0.049*	
N2	0.32677 (12)	−0.08485 (7)	0.42859 (9)	0.0378 (2)	
O1	0.20515 (10)	0.09898 (6)	0.55876 (7)	0.0424 (2)	
O2	−0.14135 (11)	0.06874 (7)	0.20344 (9)	0.0559 (3)	
O3	0.42802 (11)	−0.11268 (6)	0.51012 (9)	0.0484 (3)	
O4	0.29464 (12)	−0.12159 (6)	0.33498 (9)	0.0508 (3)	

### Atomic displacement parameters (Å^2^)

**Table:**

	*U*^11^	*U*^22^	*U*^33^	*U*^12^	*U*^13^	*U*^23^
C1	0.0473 (7)	0.0549 (8)	0.0458 (8)	0.0037 (6)	0.0177 (6)	0.0092 (6)
C2	0.0579 (8)	0.0548 (8)	0.0333 (7)	0.0044 (7)	0.0147 (6)	−0.0007 (6)
C3	0.0481 (7)	0.0391 (7)	0.0363 (6)	−0.0009 (6)	0.0092 (5)	−0.0022 (5)
C4	0.0342 (6)	0.0340 (6)	0.0318 (6)	0.0034 (5)	0.0068 (5)	0.0018 (5)
C5	0.0397 (6)	0.0417 (7)	0.0356 (6)	−0.0020 (5)	0.0069 (5)	−0.0031 (5)
C6	0.0415 (7)	0.0446 (7)	0.0511 (8)	−0.0053 (6)	0.0116 (6)	0.0026 (6)
C7	0.0853 (13)	0.0951 (15)	0.0681 (12)	−0.0151 (11)	0.0421 (10)	0.0092 (11)
C8	0.0526 (8)	0.0620 (9)	0.0371 (7)	0.0013 (7)	0.0007 (6)	−0.0017 (6)
C9	0.0604 (9)	0.0534 (9)	0.0656 (10)	0.0194 (7)	0.0253 (8)	0.0050 (7)
C10	0.0491 (7)	0.0431 (7)	0.0560 (8)	−0.0010 (6)	0.0147 (6)	0.0081 (6)
C11	0.0356 (6)	0.0322 (6)	0.0327 (6)	−0.0009 (5)	0.0069 (5)	−0.0008 (5)
C12	0.0359 (6)	0.0323 (6)	0.0345 (6)	0.0006 (5)	0.0088 (5)	0.0033 (5)
C13	0.0361 (6)	0.0335 (6)	0.0357 (6)	−0.0007 (5)	0.0090 (5)	0.0055 (5)
C14	0.0377 (6)	0.0375 (6)	0.0335 (6)	0.0027 (5)	0.0093 (5)	0.0055 (5)
C15	0.0492 (7)	0.0432 (7)	0.0375 (7)	0.0078 (6)	0.0153 (6)	0.0012 (5)
C16	0.0398 (6)	0.0398 (7)	0.0445 (7)	0.0063 (5)	0.0150 (6)	0.0048 (5)
C17	0.0321 (6)	0.0491 (7)	0.0505 (8)	0.0038 (5)	0.0102 (5)	0.0068 (6)
C18	0.0337 (6)	0.0440 (7)	0.0382 (7)	−0.0030 (5)	0.0085 (5)	0.0040 (5)
C19	0.0336 (6)	0.0360 (6)	0.0338 (6)	0.0007 (5)	0.0090 (5)	0.0030 (5)
N1	0.0419 (6)	0.0427 (6)	0.0350 (6)	0.0037 (5)	0.0041 (4)	0.0041 (4)
N2	0.0374 (5)	0.0354 (5)	0.0412 (6)	0.0004 (4)	0.0112 (4)	0.0036 (4)
O1	0.0480 (5)	0.0411 (5)	0.0337 (5)	0.0089 (4)	0.0027 (4)	−0.0012 (4)
O2	0.0455 (5)	0.0683 (7)	0.0460 (6)	0.0028 (5)	−0.0020 (4)	−0.0096 (5)
O3	0.0470 (5)	0.0467 (5)	0.0485 (6)	0.0123 (4)	0.0070 (4)	0.0074 (4)
O4	0.0571 (6)	0.0454 (5)	0.0479 (6)	0.0059 (4)	0.0099 (5)	−0.0100 (4)

### Geometric parameters (Å, º)

**Table:**

C1—C6	1.386 (2)	C10—H10B	0.9600
C1—C2	1.387 (2)	C10—H10C	0.9600
C1—C7	1.509 (2)	C11—C12	1.5030 (17)
C2—C3	1.3854 (19)	C11—C19	1.5034 (17)
C2—H2	0.9300	C11—H11	0.9800
C3—C4	1.3846 (17)	C12—N2	1.3840 (16)
C3—H3	0.9300	C12—C13	1.3915 (17)
C4—C5	1.3879 (18)	C13—N1	1.3141 (16)
C4—C11	1.5318 (16)	C13—O1	1.3557 (15)
C5—C6	1.3806 (19)	C14—C19	1.3275 (17)
C5—H5	0.9300	C14—O1	1.3857 (15)
C6—H6	0.9300	C14—C15	1.4891 (18)
C7—H7A	0.9600	C15—C16	1.5342 (18)
C7—H7B	0.9600	C15—H15A	0.9700
C7—H7C	0.9600	C15—H15B	0.9700
C8—N1	1.4528 (18)	C16—C17	1.527 (2)
C8—H8A	0.9600	C17—C18	1.5043 (19)
C8—H8B	0.9600	C17—H17A	0.9700
C8—H8C	0.9600	C17—H17B	0.9700
C9—C16	1.5281 (19)	C18—O2	1.2165 (16)
C9—H9A	0.9600	C18—C19	1.4767 (17)
C9—H9B	0.9600	N1—H1	0.8600
C9—H9C	0.9600	N2—O4	1.2421 (14)
C10—C16	1.5299 (19)	N2—O3	1.2626 (14)
C10—H10A	0.9600		
			
C6—C1—C2	117.69 (13)	C19—C11—C4	109.86 (10)
C6—C1—C7	120.63 (15)	C12—C11—H11	108.7
C2—C1—C7	121.66 (15)	C19—C11—H11	108.7
C3—C2—C1	121.24 (13)	C4—C11—H11	108.7
C3—C2—H2	119.4	N2—C12—C13	119.94 (11)
C1—C2—H2	119.4	N2—C12—C11	117.14 (11)
C4—C3—C2	120.73 (13)	C13—C12—C11	122.74 (11)
C4—C3—H3	119.6	N1—C13—O1	112.05 (11)
C2—C3—H3	119.6	N1—C13—C12	127.95 (12)
C3—C4—C5	118.18 (12)	O1—C13—C12	120.00 (11)
C3—C4—C11	121.74 (11)	C19—C14—O1	122.61 (11)
C5—C4—C11	120.07 (11)	C19—C14—C15	126.15 (12)
C6—C5—C4	120.87 (12)	O1—C14—C15	111.22 (11)
C6—C5—H5	119.6	C14—C15—C16	111.57 (11)
C4—C5—H5	119.6	C14—C15—H15A	109.3
C5—C6—C1	121.27 (13)	C16—C15—H15A	109.3
C5—C6—H6	119.4	C14—C15—H15B	109.3
C1—C6—H6	119.4	C16—C15—H15B	109.3
C1—C7—H7A	109.5	H15A—C15—H15B	108.0
C1—C7—H7B	109.5	C17—C16—C9	109.58 (12)
H7A—C7—H7B	109.5	C17—C16—C10	109.86 (11)
C1—C7—H7C	109.5	C9—C16—C10	109.80 (12)
H7A—C7—H7C	109.5	C17—C16—C15	108.66 (11)
H7B—C7—H7C	109.5	C9—C16—C15	108.98 (11)
N1—C8—H8A	109.5	C10—C16—C15	109.95 (11)
N1—C8—H8B	109.5	C18—C17—C16	115.30 (10)
H8A—C8—H8B	109.5	C18—C17—H17A	108.5
N1—C8—H8C	109.5	C16—C17—H17A	108.5
H8A—C8—H8C	109.5	C18—C17—H17B	108.5
H8B—C8—H8C	109.5	C16—C17—H17B	108.5
C16—C9—H9A	109.5	H17A—C17—H17B	107.5
C16—C9—H9B	109.5	O2—C18—C19	120.16 (12)
H9A—C9—H9B	109.5	O2—C18—C17	122.13 (12)
C16—C9—H9C	109.5	C19—C18—C17	117.67 (11)
H9A—C9—H9C	109.5	C14—C19—C18	118.79 (12)
H9B—C9—H9C	109.5	C14—C19—C11	122.48 (11)
C16—C10—H10A	109.5	C18—C19—C11	118.69 (11)
C16—C10—H10B	109.5	C13—N1—C8	124.91 (12)
H10A—C10—H10B	109.5	C13—N1—H1	117.5
C16—C10—H10C	109.5	C8—N1—H1	117.5
H10A—C10—H10C	109.5	O4—N2—O3	120.52 (11)
H10B—C10—H10C	109.5	O4—N2—C12	118.63 (11)
C12—C11—C19	108.85 (10)	O3—N2—C12	120.85 (11)
C12—C11—C4	111.92 (10)	C13—O1—C14	119.87 (10)
			
C6—C1—C2—C3	−0.1 (2)	C9—C16—C17—C18	−169.54 (12)
C7—C1—C2—C3	178.62 (16)	C10—C16—C17—C18	69.75 (15)
C1—C2—C3—C4	−1.1 (2)	C15—C16—C17—C18	−50.56 (15)
C2—C3—C4—C5	1.85 (19)	C16—C17—C18—O2	−157.41 (13)
C2—C3—C4—C11	−177.78 (12)	C16—C17—C18—C19	24.66 (17)
C3—C4—C5—C6	−1.36 (19)	O1—C14—C19—C18	176.46 (11)
C11—C4—C5—C6	178.27 (12)	C15—C14—C19—C18	−5.1 (2)
C4—C5—C6—C1	0.2 (2)	O1—C14—C19—C11	−6.08 (19)
C2—C1—C6—C5	0.6 (2)	C15—C14—C19—C11	172.31 (12)
C7—C1—C6—C5	−178.14 (16)	O2—C18—C19—C14	−173.35 (13)
C3—C4—C11—C12	119.44 (13)	C17—C18—C19—C14	4.61 (18)
C5—C4—C11—C12	−60.18 (15)	O2—C18—C19—C11	9.09 (18)
C3—C4—C11—C19	−119.52 (12)	C17—C18—C19—C11	−172.94 (11)
C5—C4—C11—C19	60.86 (14)	C12—C11—C19—C14	18.29 (16)
C19—C11—C12—N2	166.69 (10)	C4—C11—C19—C14	−104.58 (13)
C4—C11—C12—N2	−71.68 (13)	C12—C11—C19—C18	−164.25 (10)
C19—C11—C12—C13	−18.20 (16)	C4—C11—C19—C18	72.88 (13)
C4—C11—C12—C13	103.42 (13)	O1—C13—N1—C8	1.70 (18)
N2—C12—C13—N1	0.7 (2)	C12—C13—N1—C8	−178.21 (13)
C11—C12—C13—N1	−174.26 (12)	C13—C12—N2—O4	179.94 (11)
N2—C12—C13—O1	−179.19 (11)	C11—C12—N2—O4	−4.82 (16)
C11—C12—C13—O1	5.84 (18)	C13—C12—N2—O3	0.15 (18)
C19—C14—C15—C16	−23.05 (19)	C11—C12—N2—O3	175.40 (11)
O1—C14—C15—C16	155.49 (11)	N1—C13—O1—C14	−170.87 (11)
C14—C15—C16—C17	48.46 (15)	C12—C13—O1—C14	9.05 (17)
C14—C15—C16—C9	167.81 (12)	C19—C14—O1—C13	−9.14 (18)
C14—C15—C16—C10	−71.80 (15)	C15—C14—O1—C13	172.26 (11)

### Hydrogen-bond geometry (Å, º)

**Table:**

*D*—H···*A*	*D*—H	H···*A*	*D*···*A*	*D*—H···*A*
N1—H1···O3	0.86	1.98	2.6024 (15)	129
C5—H5···O3^i^	0.93	2.58	3.0685 (17)	113
C10—H10*A*···O4^ii^	0.96	2.54	3.4335 (19)	154

Symmetry codes: (i) −*x*+1, −*y*, −*z*+1; (ii) −*x*, *y*+1/2, −*z*+1/2.
